# Functional proteins in breast milk and their correlation with the development of the infant gut microbiota: a study of mother-infant pairs

**DOI:** 10.3389/fmicb.2023.1239501

**Published:** 2023-09-13

**Authors:** Menglu Xi, Dong Liang, Yalu Yan, Sufang Duan, Houxi Leng, Haibing Yang, Xiaojin Shi, Xiaona Na, Yucheng Yang, Celi Yang, Ignatius Man-Yau Szeto, Ai Zhao

**Affiliations:** ^1^Vanke School of Public Health, Tsinghua University, Beijing, China; ^2^China National Center for Food Safety Risk Assessment, Beijing, China; ^3^Inner Mongolia Yili Industrial Group, Co. Ltd., Yili Maternal and Infant Nutrition Institute (YMINI), Beijing, China; ^4^Inner Mongolia Dairy Technology Research Institute Co. Ltd., Hohhot, China; ^5^National Center of Technology Innovation for Dairy, Hohhot, China

**Keywords:** protein, breast milk, 16S rRNA sequencing, gut microbiota, infant

## Abstract

**Introduction:**

Proteins in breast milk play an important role in the growth and development of infants. This study aims to explore the correlation between functional proteins in breast milk and the infant gut microbiota.

**Methods:**

Twenty-three mothers and their infants were enrolled and breast milk samples and infant fecal samples were collected. Breast milk protein content was determined by UPLC-MS/MS, and 16S rRNA sequencing was employed to analyze the gut microbiota of infant.

**Results:**

The results indicated that the secretory immunoglobulin A (sIgA) content in breast milk was positively correlated with the abundance of *Veillonella parvula*. The κ-casein content was positively correlated with the abundance of *Clostridium butyricum*. The osteopontin (OPN) and lactalbumin contents were positively correlated with the abundance of *Parabacteroides distasonis* at 42 days. Functional pathway analysis showed that the OPN and κ-casein contents in breast milk were significantly correlated with amino acid, pyruvate, propionic acid, linoleic acid, and alpha-linolenic acid metabolic pathways in early life.

**Discussion:**

The results of this study suggest that specific proteins in breast milk can influence the abundance of certain gut microbes in infants, playing an important role in early immune and metabolic development.

## Introduction

Human breast milk is the most ideal natural food for infants (Yi and Kim, 2021). In addition to providing energy, breast milk contains a variety of bioactive compounds, which are also considered to be important factors in the start-up and development of the newborn gut microbiota (Lyons et al., 2020; Nuzzi et al., 2021). However, there are significant individual differences of breast milk components. There are studies that report the importance of breastfeeding being influenced by the mother’s diet and then modulate gut microbiome of infants (Manoppo et al., 2022; Xi et al., 2022).

Protein is one of the three important macronutrients in breast milk and falls into three categories: casein (β-casein, κ-casein, and αs1-casein), whey protein [lactoferrin (LF), alpha-lactoalbumin, secretory immunoglobulin A (sIgA), and serum albumin], and milk fat globule membrane protein (Andreas et al., 2015). Casein and whey proteins represent the main breast milk proteins, which provide nutrients for infant growth and development and have multiple biological functions, such as anti-infection, inhibiting the expression of inflammatory factors, and establishing an autoimmune system (Bumrungpert et al., 2018; Kim et al., 2021). It is well documented that the immune functions are regulated by the gut microbiome. Gut bacteria can regulate the expression of inflammatory cytokines and immune system through the release of metabolites (Brooks et al., 2023; Taitz et al., 2023). Recent studies have also demonstrated the potential regulatory effects of proteins on the infant gut microbiota. Mastromarino et al. measured the quantity of *Bifidobacterium* and *Lactobacillus* in infant feces by real-time polymerase chain reaction (PCR) and found that these microbes in infant feces were closely related to the concentration of fecal LF (Mastromarino et al., 2014). Brück et al. investigated the effect of infant formula rich in bovine α-lactoalbumin and casein glycopeptide (CGMP) on the fecal microbiota of healthy full-term infants and reported that α-lactoalbumin and CGMP may promote intestinal microbiota composition (Brück et al., 2006). Certain microbiota, such as *Bifidobacterium* and *Lactobacillus* mentioned above, can down-regulate the expression of NF-κB gene, up-regulate MUC2 and other intestinal mucosal barrier factors, reduce intestinal inflammation and improve intestinal structure and morphology, which could further explain the protein’s immune function (Fang et al., 2023; Mishima et al., 2023). However, to the best of knowledge, no study had explored the correlation between breastmilk proteins’ contents and infants’ gut microbiota.

Of note, proteins level in human milk varies among individuals (Liu et al., 2019). With the extension of the lactation period, the level of certain proteins in breast milk decrease with time (say, from 42 days to 3 months), whereas others showed an increasing trend (Verd et al., 2018). It would also be interesting to know the dynamic effects of different proteins on the infant gut microbiota. Therefore, we employed a prospective design to evaluate the functional protein profiles of breast milk and the infant gut microbiota at 42 days and 3 months postpartum, respectively. We aimed to examine the correlation between milk protein levels and the infant gut microbiota and to explore the effect time window.

## Materials and methods

### Participants

**Figure 1:**
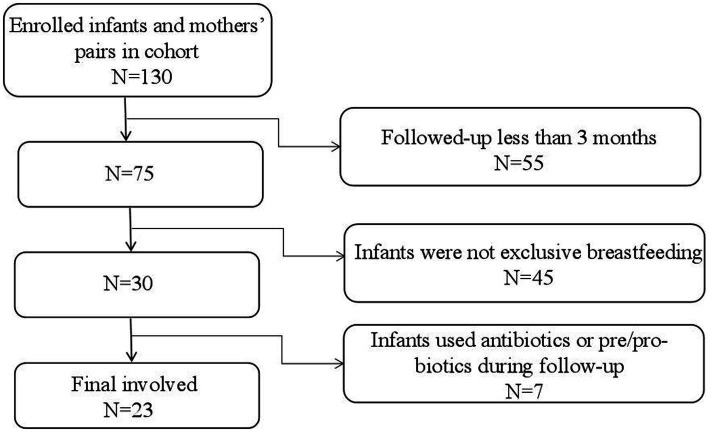
Flow chart of data selection.

Data for the participants were obtained from a birth cohort of the “Chinese Maternal and Infants Nutrition and Health Study”, which was conducted in four Chinese cities (Chenzhou, Lanzhou, Cangzhou, and Xuchang). This cohort enrolled lactating mothers and their infants after delivery (within 3 days postpartum) and followed them for up to 1 year postpartum. Breast milk and feces from the infants were collected at certain observational points. In this study, samples and data of 23 infants and their lactating mothers at 42 days and 3 months postpartum were used. The 42 days postpartum was chosen for this study because the first time of regular postnatal health check-up was required at this time (Cheng et al., 2023). When design the cohort, we hoped every follow-up could be accompanied with regular health check-ups. In addition, previously studies have confirmed that it usually takes 42 days for postpartum women’s endometrium to fully heal and recover (Cai et al., 2020; Cheng et al., 2023). The 3 months was also another time point for regular health check-ups for infants. And it is also a time when breast milk is considered the optimal full food for infants and it is a crucial time for infants’ gut microbiota development. After 4 months, the gut microbiota will greatly be influenced by the introducing of complementary food (Ma et al., 2022). The inclusion criteria for this study were: (1) healthy singleton, (2) full-term delivery (37–42 weeks), (3) exclusive breastfeeding, (4) no disability or disease diagnosed at birth, and (5) infants’ Apgar score ≥ 8, (6) mothers without gastric and intestinal illnesses and nipple or lacteal gland diseases. In addition, infants who had used any antibiotics or any products contain prebiotics or probiotics during the follow-up and mothers who had used any antibiotics in the last four weeks prior to the enrollment or during the follow-up were further excluded from the current analysis. By the time of this study was carried out, a total of 130 pairs of infants and mothers were successfully enrolled in the cohort, and 75 of infants and their lactating mothers were followed up for at least 3 months. Out of them, 30 infants were exclusive breastfed and 7 pairs did not meet the criteria. Finally, samples and data of 23 pairs of infants and mothers were used for this study (Figure 1). All participants gave written informed consent. The protocol was approved by the Ethics Committee of Tsinghua University (project no.20210126).

### Data collection

A face-to-face interviewer-administered questionnaire was used to collect maternal and child health information by trained health care professionals during regular postnatal health check-ups. There were four parts in the questionnaire, including socio-demographic characteristics (age at pregnancy and education level), lifestyle (smoking status and alcohol intake), pregnancy information (mode of delivery, infant gender, birth weight, birth length, head circumference, etc.), and delivery history. The height and weight of the lactating women were measured by trained health care professionals during their visits to the maternity and child hospital. The postpartum weight retention was calculated as the current weight minus the self-reported pre-pregnancy weight.

To avoid the influence of relevant factors on the composition of breast milk, standardized breast milk sampling was adopted for all subjects in this study. Lactating mothers were instructed to feed their infants and empty their breasts between 6:00 and 7:00 a.m., and breast milk was collected at the second feeding time in the morning (9:00–11:00 a.m.). All milk from one breast was collected with a sterile milk aspirator. The foremilk, middle milk, and hindmilk were gently mixed and then immediately frozen at −80°C. For fecal sample collection, lactating mothers were required to use aseptic collection tubes to collect 3 g of infant stool, and then delivere them to the laboratory with ice bags. Samples were stored at −80°C.

### Breast milk protein analysis

The Bradford method was used to detect the total protein content in human milk. The specific operations were as follows: First, the 5 mg Coomassie blue reagent was dissolved in 25 mL of 95% ethanol, mixed with 50 mL of 85% phosphoric acid, and distilled water was added to bring the volume to 500 mL, the final reagent contains 0.01% (W/V) Coomassie brilliant blue (Bio-Rad, United States). Then, the diluted human milk sample was added to the aforementioned Coomassie Blue reagent. The absorbance was measured at 595 nm (R-Biopharm WELL Reader, R-Biopharm, Germany).

The concentrations of LF, α-lactoalbumin, serum albumin, immunoglobulin A, OPN, β-casein, αs1-casein, and κ-casein in breast milk samples were quantitatively determined by the following method (Zhang et al., 2023). First, 0.5 g of human milk sample was diluted to 10 mL with water. Then, 0.1 mL of the diluted sample was transferred to an Eppendorf tube containing 10 μL of internal standard [the target characteristic peptide segment labeled by the isotope, which were purchased from ChinaPeptides Co. Ltd. (Shanghai, China)], 10 μL of dithiothreitol solution, and 845 μL of water. Next, 10 μL of iodoacetamide solution (54 mg/mL in water) was added, and the solution was incubated in the dark for 30 min at room temperature. Breast milk proteins were digested with trypsin at 37°C for 4 h; the reaction was terminated with formic acid (Chen et al., 2016).

The peptides were analyzed with ultra-performance liquid chromatography (Waters UPLC I-Class, Waters, United States)–tandem mass spectrometry (XEVO TQ-XS, Waters, United States). The specific operations for drawing standard curves for different concentrations of target proteins were carried out as follows: accurately absorbed the standard mixed solution of different volumes of characteristic peptide segments, added the internal standard mixed solution of the same volume, and adjusted the volume of 0.1% formic acid solution to 1 mL to obtain the standard series of working fluids with concentrations of 10, 20, 40, 60, 80, and 100 μg/L, respectively, for analysis by ultra-high performance liquid chromatography in tandem with a quadrupole mass spectrometer. Each concentration point of the standard curve is prepared by the standard mixed intermediate solution according to the proportion. For example, each characteristic peptide segment is prepared into a 1 mg/mL reserve solution, and then a mixed intermediate solution with a concentration of 10 μg/mL is prepared from the reserve solution, and then 10, 25, 50, 100, 200, 300, and 400 μL mixed intermediate solution are removed, respectively. The standard series of working solutions with a concentration of 0.1, 0.25, 0.5, 1.0, 2.0, 3.0, and 4.0 μg/mL are obtained by holding water to 1 mL. It was calculated by Masslynx quantitative software and quantified by the internal standard method. The Acquire BEH300 C18 column (300 Å, 3.5 μm, 2.1 mm × 150 mm) was used. Mobile phase A was 0.1% formic acid and mobile phase B was an acetonitrile solution of 0.1% formic acid. The flow rate was 0.3 mL/min. The following gradient was used: 3% B to 32% B within 0–5 min; increase to 100% B within 0.1 min, and maintain it for 1 min; then decrease to 3% B within 0.1 min, and maintain it for 1.8 min. The column temperature was 40°C.

### DNA extraction from infant feces and bioinformatics analysis of 16S ribosomal RNA (rRNA)

The QIAamp DNA Stool Mini Kit was used to extract DNA from feces (150 mg), and the 16S V3–V4 region was sequenced bi-directionally on the Illumina high-throughput platform. PCR amplification was performed using the ABI9700 PCR instrument (ABI, Thermo Fisher Scientific, United States), and the reagents used include Phusion ultra fidelity DNA polymerase (NEB, United States). The primers were 357F (5′- ACTCCTACGGRAGGCAG-3′) and 806R (5′- GGACTACHVGGGTWTCTAAT-3′), and two-step PCR amplification was performed. The first PCR reaction system was as follows: 5×Buffer 10 μL, dNTP (10 mM) 1 μL, Phusion 1 U, forward and reverse outer primers (10uM) 1 μL each, template DNA 20–50 ng, and ultra-pure water to 50 μL. The PCR reaction condition was 94°C for 2 min. 94°C for 30 s, 56°C for 30 s, 72°C for 30 s, 72°C for 5 min, 24 cycles. The second PCR reaction system was: 5×Buffer 8 μL, dNTP (10 mM) 1 μL, Phusion ultra-fidelity DNA polymerase 0.8 U, forward and reverse outer primers (10uM) 1 μL each, template DNA 5 μL, and ultra-pure water was added to 40 μL. The PCR reaction condition was 94°C for 2 min. 94°C for 30 s, 56°C for 30 s, 72°C for 30 s, 72°C for 5 min, 10°C for 8 cycles. The products were recovered using the Axygen Gel Recovery Kit (Axygen, United States) from the gel.

All PCR products were fluorescence quantified using the FTC-3000TM Real-Time PCR instrument (Fengling Shanghai, China), mixed with an equal molar ratio, then amplified by an 8-cycle PCR (same method as above). The connectors, sequencing primers and barcodes required by Illumina platform were added to both ends of the target fragment to complete library construction. The constructed library was sequenced in Microbased Biotechnology Co., Ltd. (Shanghai, China) by using Novaseq 6,000 SP 500 Cycle Reagent Kit (Illumina, United States). The reagents used were TB Green Premix Ex Taq (Takara, Japan). Primers use adaptor sequences. Equipment for sequencing: illumina novaseq (Illumina, United States). The Kit for the library preparation is the Novaseq 6,000 SP 500 Cycle Reagent Kit (Illumina, United States).

Reads obtained from sequencing distinguish each sample according to the barcode, and quality control and filtering were conducted. Trimmatic (version 0.38) software was used to remove low-quality sequences, and cutadapt (version 1.16) software was used to process sequencing connectors and primers to obtain optimized sequences. USEARCH software was used to cluster the processed CleanTags into operational taxonomic units (OTUs) at 97% similarity. The species classification was completed based on OTU annotation. Kyoto Encyclopedia of Genes and Genomes (KEGG) is a comprehensive database on gene function annotation, including gene function, classification, metabolic pathways, and other information. KEGG Orthology (KO) is used to classify each gene with a known function and its homologous genes into one group. Based on the assumption that homologous genes have similar functions, homologous genes are considered to be orthologous, and the function of this gene is used as the function of this analysis. We used KEGG analysis to explore the possible functions of certain microbes regulated by target breast milk proteins. The nonparametric Wilcoxon test was used to compare the significant differences among proteins, and the related differences of KO were further screened. The datasets used in this study have been uploaded to the NCBI repository with the entry number SRP387978: PRJNA858926.

### Statistical analysis

R 3.6.2 (R Development Core Team, Vienna, Austria) was used for statistical analysis. Continuous variables are presented as the mean ± standard deviation (SD) or median [25th percentile (P25), 75th percentile (P75)], and categorical variables are presented as the proportion or composition ratio. The breast milk protein levels were grouped into high and low groups according to their median. Significant differences between groups were analyzed by the nonparametric Wilcoxon test. Spearman’s rank correlation was used to analyze the correlation between each breast milk protein and the infant gut microbiota. The partial correlation analysis was performed using the ppcor package with the delivery modes adjusted. A *p*-value <0.05 was considered significantly different.

## Results

### Characteristics of the participants

Table 1 provides the basic information of the 23-lactating mother–infant pairs. No mother reported smoking or drinking. The average ± SD maternal age was 30.2 ± 4.5 years. The majority of the women in this study had experienced their first parity. Overall, 43.4% of lactating mothers had high weight retention (>5 kg) at 3 months after delivery. BMI ranges before pregnancy from 18.7 to 25.8. The average birth weight of the infants was 3,305 (3,000, 3,525) g. Among them, the majority of babies were born by vaginal delivery, and male infants accounted for 56.5%. The mean gestational age of the infants was 39.0 ± 1.0 weeks.

**Table 1:** Basic characteristics of participants.

Characteristics		Description*
*N* = 23 Mother		
Age (years)		30.2 ± 4.5
Parity	First	18 (78.3)
	Second or above	5 (21.7)
BMI before pregnancy (kg/m^2^)		20.1 ± 2.2
BMI at 3 m (kg/m^2^)		22.1 ± 2.4
Postpartum weight retention at 3 m(kg)	<3	7 (30.4)
	3–5	6 (26.1)
	>5	10 (43.4)
Education level	Senior high school or below	7 (30.4)
	Junior college degree	10 (43.5)
	Bachelor’s degree or above	6 (26.1)
Infant		
Birth weight (g)		3,305 (3,000, 3,525)
Gestational age (weeks)		39.0 ± 1.0
Gender	Male	13 (56.5)
	Female	10 (43.5)
Mode of delivery	Vaginal delivery	15 (65.2)
	Cesarean section	8 (34.8)

*Continuous variables were presented as the Mean ± SD or median (P25, P75) according to normality of data. Categorical variables were presented as *N* (%). Body mass index (BMI) = weight (kg) / height (m) squared.

### Proteins in breast milk

With UPLC-MS/MS, nine proteins were analyzed in human milk at 42 days and 3 months postpartum. The total protein content was 1574.13 ± 229.93 mg/100 g breast milk at 42 days and 1241.20 ± 239.43 mg/100 g breast milk at 3 months. The most abundant protein was casein, followed by α-lactalbumin and LF. Except for sIgA, the content of the other proteins in breast milk was significantly higher at 42 days than at 3 months (Table 2). We also further analyzed the protein content in breast milk with different delivery modes or different postpartum weight retention, and the results were presented in Supplementary Table S1 of supplementary materials. It was found that there was no significant difference in protein content in breast milk with different delivery modes or different postpartum weight retention.

**Table 2:** Proteins content in breast milk at 42 days and 3 months postpartum.

Protein (mg/100 g)	42d		3 m		*p*-value*
	Mean ± SD	P50 (P25, P75)	Mean ± SD	P50 (P25, P75)	
Lactoferrin	99.42 ± 30.99	99.48 (82.94, 116.23)	75.25 ± 28.07	69.44 (57.76, 97.21)	<0.01
Albumin	28.3 ± 6.02	27.82 (23.11, 32.33)	23.73 ± 6.32	23.64 (21.07, 25.45)	<0.05
α-Lactalbumin	380.27 ± 46.80	381.24 (340.14, 419.92)	272.75 ± 53.68	284.37 (240.04, 304.62)	<0.01
Osteopontin	24.28 ± 5.02	26.01 (20.84, 27.7)	18.26 ± 3.95	18.45 (15.16, 21.23)	<0.01
SIgA	38.65 ± 13.37	39.99 (25.78, 47.22)	33.04 ± 13.66	32.83 (22.78, 41.52)	0.1539
αs1-Casei	79.85 ± 14.39	81.45 (73.60, 87.32)	59.79 ± 13.30	58.36 (54.00, 68.98)	<0.01
β-Casein	390.9 ± 81.99	400.88 (350.81, 450.05)	326.47 ± 80.99	332.48 (296.58, 387.80)	<0.01
κ-Casein	31.33 ± 6.05	30.22 (26.99, 34.39)	23.64 ± 5.57	24.49 (20.41, 26.80)	<0.01
Casein	501.13 ± 92.58	511.25 (445.06, 551.35)	408.26 ± 93.56	413.89 (364.96, 485.98)	<0.01
Total	1574.13 ± 229.93	1594.67 (1429.86, 1742.31)	1241.20 ± 239.43	1242.00 (1116.44,1405.53)	<0.01

*Significant differences between groups were analyzed by Wilcox test.

### Correlation between human milk protein and the gut microbiota of infant

According to the composition of the gut microbiota at the genus level at 42 days and 3 months (Supplementary Figure S1), the intestinal microbiota of infants was mainly composed of the genera *Bifidobacterium, Escherichia, Streptococcus,* and *Enterobacter*. The most abundant intestinal microorganisms were *Escherichia coli, Bifidobacterium longum, Streptococcus salivarius, Enterobacter aerogenes, Lactobacillus gasseri, and Bifidobacterium pseudocatenulatum*.

**Figure 2:**
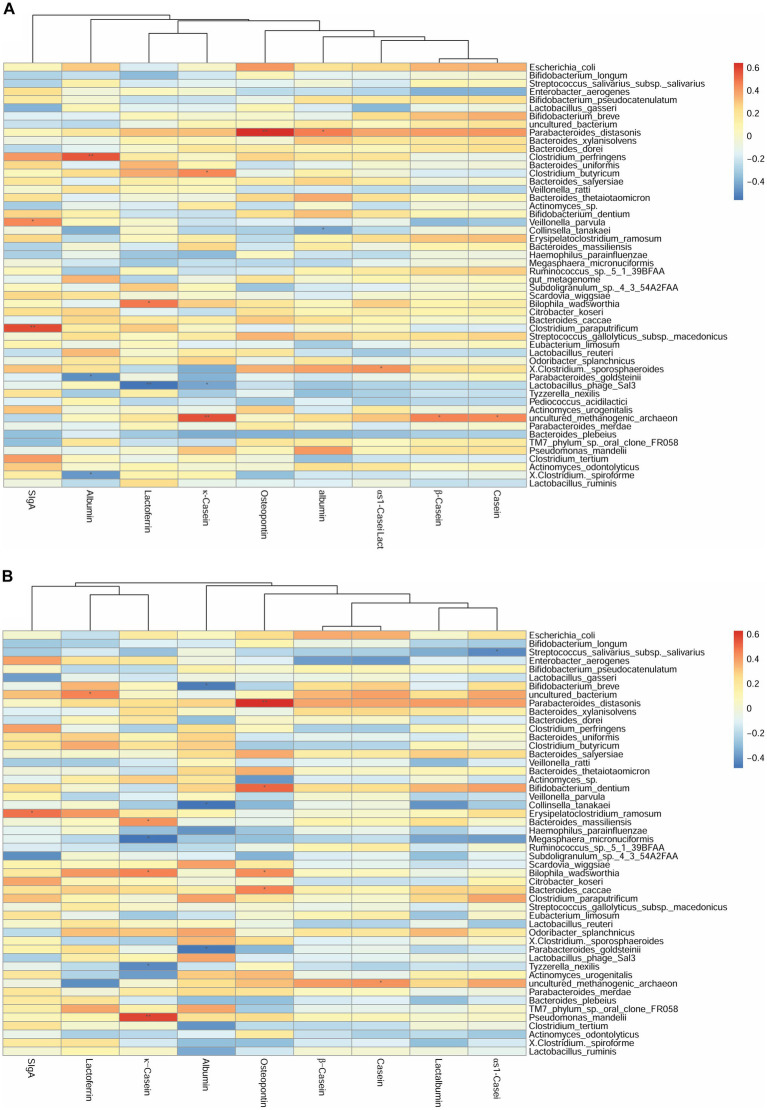

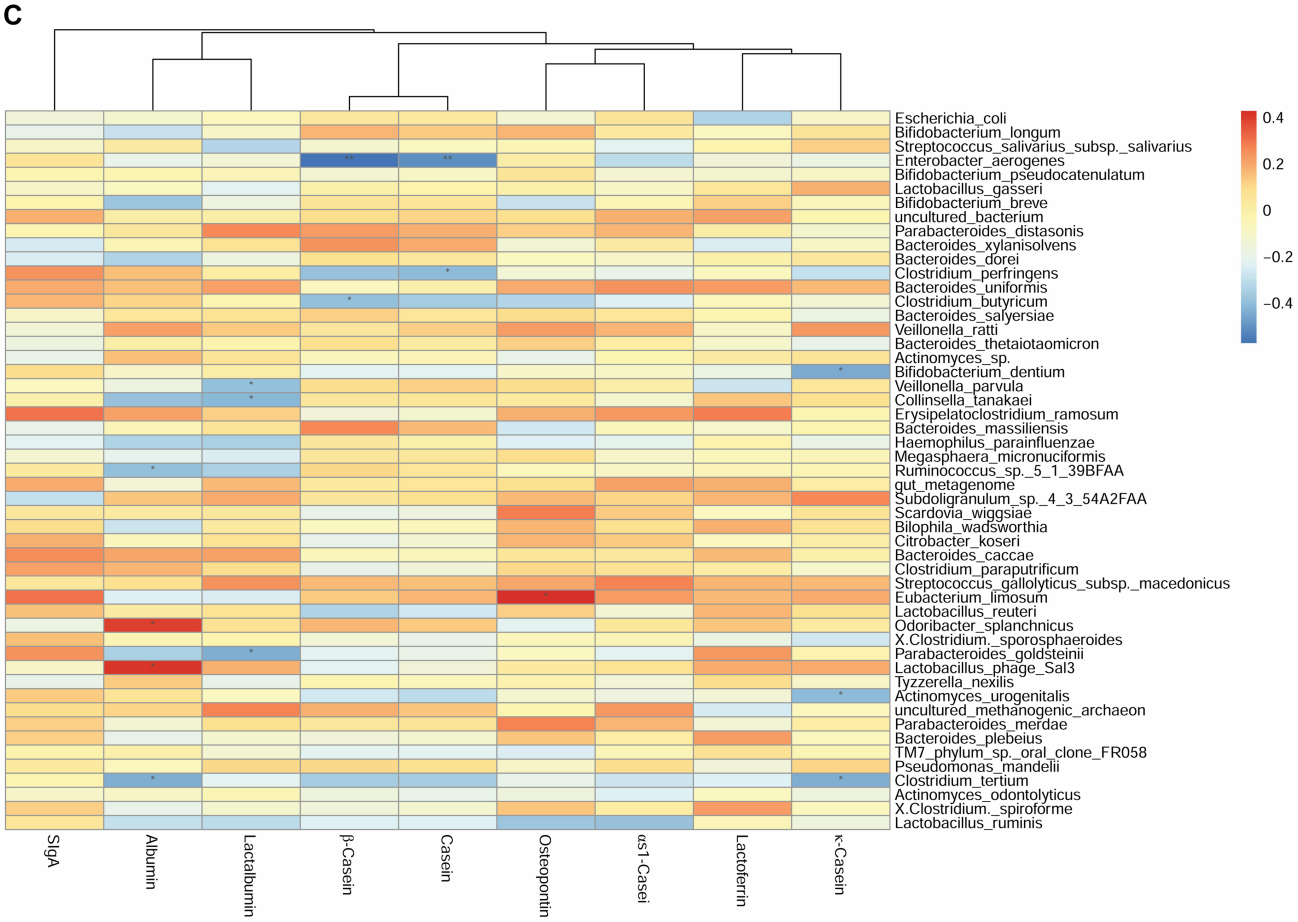
Correlation heatmap of breast milk proteins with the top 40 most-abundant infant gut microbiota at the species level based on Spearman analysis. The panels represent **(A)** breast milk proteins and infant feces at 42 days, **(B)** breast milk proteins at 42 days and infant feces at 3 months, and **(C)** breast milk proteins and infant feces at 3 months. The horizontal axis represents the nine proteins detected in breast milk, and the vertical axis represents the top 40 most-abundant infant gut microbiota at the species level in infant’s gut microbiota. The color represents the r-value of the correlation coefficient (unadjusted), red represents the positive correlation, blue represents the negative correlation, and the darker means a higher *r*-value, *p < 0.05.

We first investigated the correlation between human milk protein and the top 40 most-abundant infant intestinal microbiota at the genus level (Supplementary Figure S2) and species level (Figure 2) respectively. For genus level, at 42 days, the sIgA content of breast milk was positively correlated with the abundance of *Veillonella* (*r* = 0.42, *p* < 0.05). The LF content was negatively correlated with the abundance of *Bifidobacterium* (*r* = −0.44, *p* < 0.05). The OPN content was positively correlated with the abundance of *Parabacteroides* (*r* = 0.63, *p* < 0.01). Considering the association between breast milk protein content at 42 days and the infant gut microbiota at 3 months, the OPN content was positively correlated with the abundance of *Parabacteroides* (*r* = 0.62, *p* < 0.01). Casein and lactalbumin were positively correlated with *Atopobium* (*r* = 0.49, *p* < 0.05) and *Alistipes* (*r* = 0.43, *p* < 0.05), respectively. Finally, at 3 months, the OPN content was positively correlated with the abundance of *Eubacterium* (*r* = 0.43, *p* < 0.05).

For the species level, at 42 days, the SIgA content was positively correlated with the abundance of *Veillonella parvula* (*r* = 0.45, *p* < 0.05). The κ-casein content was positively correlated with the abundance of *Clostridium butyricum* (*r* = 0.44, *p* < 0.05). The OPN and lactalbumin contents were positively correlated with the abundance of *Parabacteroides distasonis* (*r* = 0.64, *p* < 0.01). The albumin content was negatively correlated with the abundance of *Parabacteroides goldsteinii* (*r* = −0.49, *p* < 0.05) (Figure 2A). Considering the breast milk protein content at 42 days and the infant gut microbiota at 3 months, the sIgA content was negatively correlated with the abundance of *Erysipelatoclostridium ramosum* (*r* = 0.49, *p* < 0.05). The OPN content was positively correlated with the abundance of *P. distasonis* (*r* = 0.63, *p* < 0.01) and *Bifidobacterium dentium* (*r* = 0.51, *p* < 0.05) (Figure 2B). Finally, at 3 months, the OPN content was positively correlated with the abundance of *Eubacterium limosum* (*r* = 0.43, *p* < 0.05). The κ-casein content was negatively correlated with the abundance of *B. dentium* (*r* = −0.44, *p* < 0.05) (Figure 2C).

The correlation between breast milk protein and specific probiotics such as *Bifidobacterium* and *Lactobacillus* were further explored (Supplementary Figure S3); there were no additional significant correlations. Partial correlation analyses were done to repeat the above correlations after adjusting for the delivery mode, and there were no changes (Supplementary Figure S4).

### Functional pathways

Based on the above correlation analysis of breast milk protein contents and the gut microbiota at the species level, we screened OPN, κ-casein, and sIgA for their effects on the infant gut microbiota. Then, we further explored functional expression. We used KEGG to classify all levels of metabolic pathways. The 23 infants’ microbiotas corresponded to six first-level, 40 s level, and 324 third-level metabolic pathways. We attempted to analyze 180 out of 324 tertiary metabolic pathways under 15 KEGG secondary metabolic pathways, which are highly related to early health and development.

**Figure 3:**
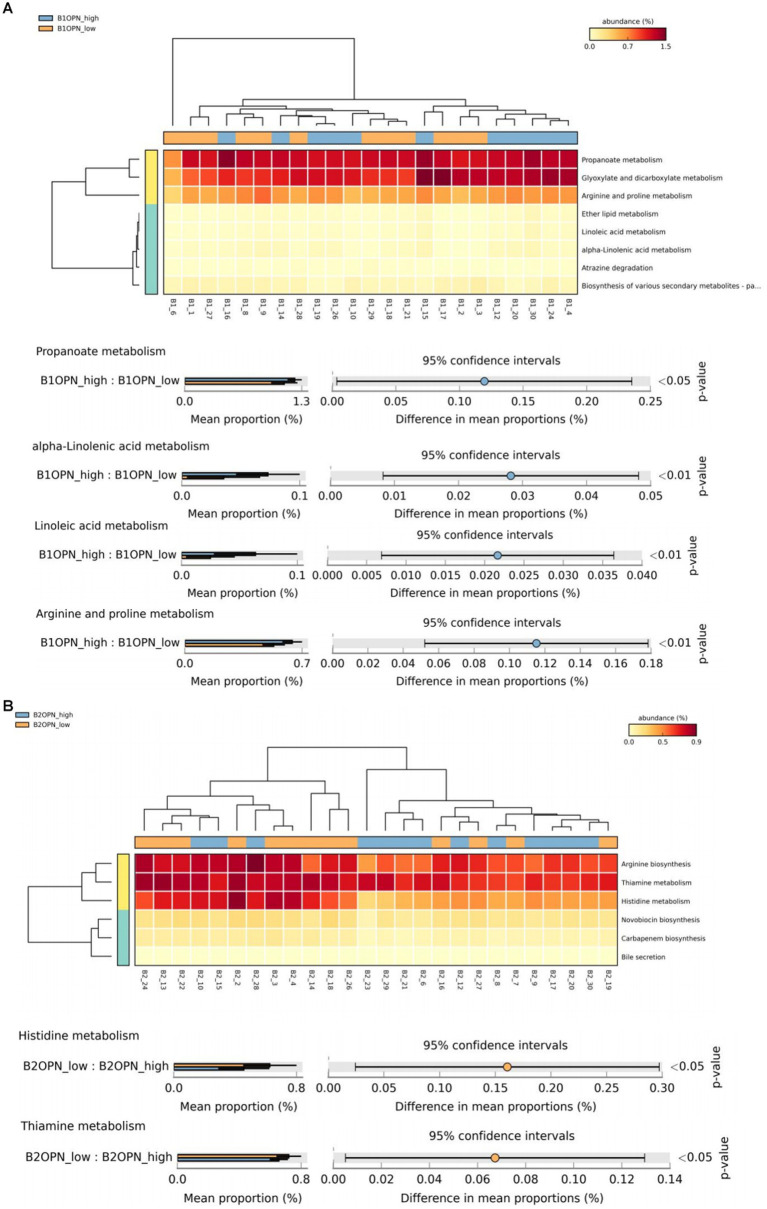

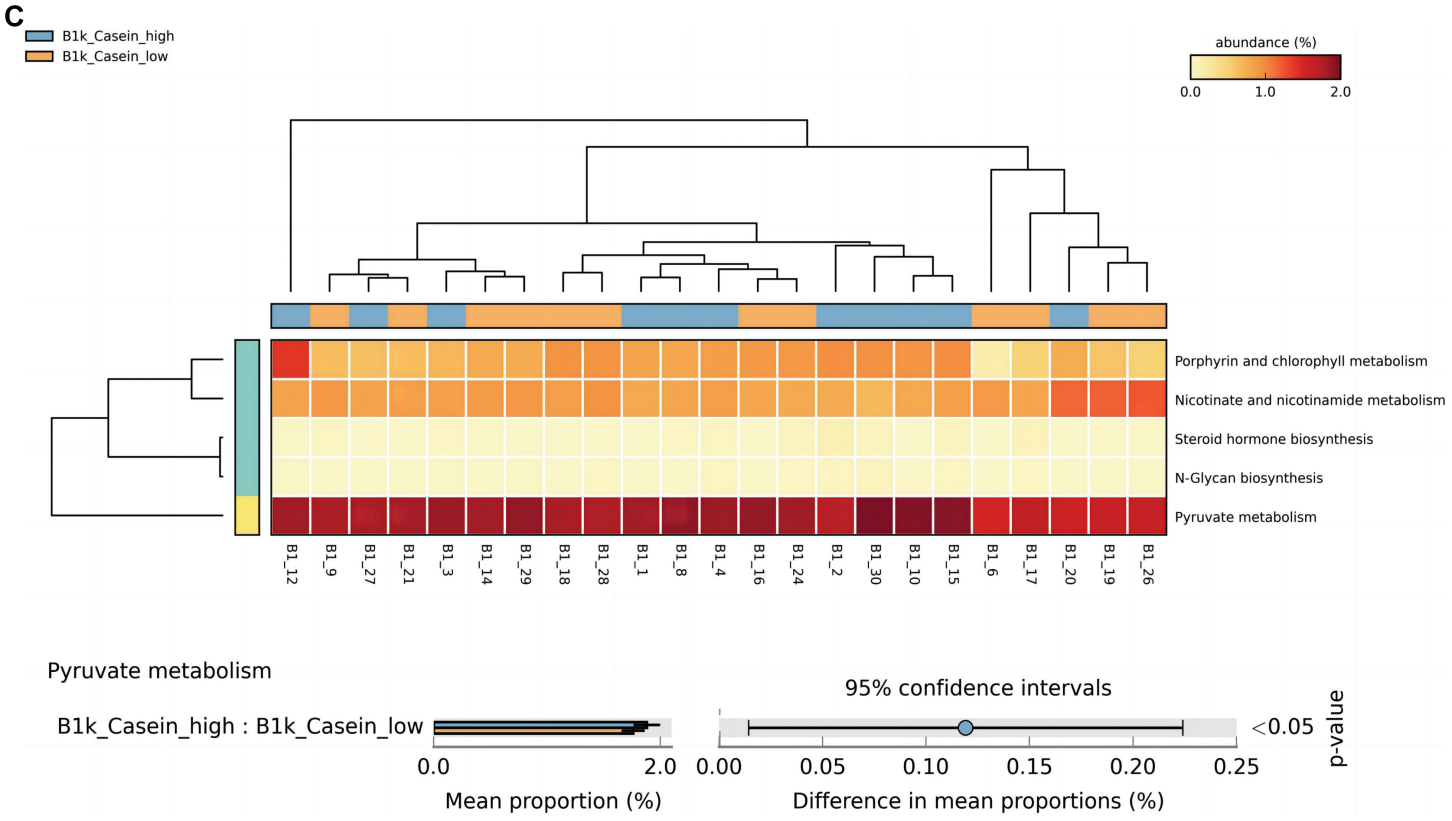
Analysis of specific breast milk proteins and Kyoto Encyclopedia of Genes and Genomes (KEGG) tertiary metabolic pathways in human breast milk. The panels represent the KEGG tertiary pathway enrichment of breast milk osteopontin (OPN) at **(A)** 42 days and **(B)** 3 months, and **(C)** κ-casein at 42 days in the low content (orange) and high content (green) groups. The KEGG metabolic pathway levels of different protein groups were compared by non-parametric Wilcoxon test. The horizontal coordinate represents the sample information detected. Yellow to red indicates the abundance distribution of different samples in tertiary metabolic pathways from low to high. The lower bar represents the tertiary metabolic pathways with significant differences. A *p*-value <0.05 was considered significantly different.

At 42 days, there were significant differences in alpha-linolenic acid, linoleic acid, arginine and proline metabolism, and propanoate metabolism between the high and low OPN content groups (Figure 3A). At 3 months, there were significant differences in histidine and thiamine metabolism between the high and low OPN content groups (Figure 3B). The corresponding metabolic pathway for κ-casein at 42 days was pyruvate metabolism (Figure 3C). There was no significant difference in the metabolic pathway between the high and low κ-casein and SIgA content groups at 3 months.

**Figure 4:**
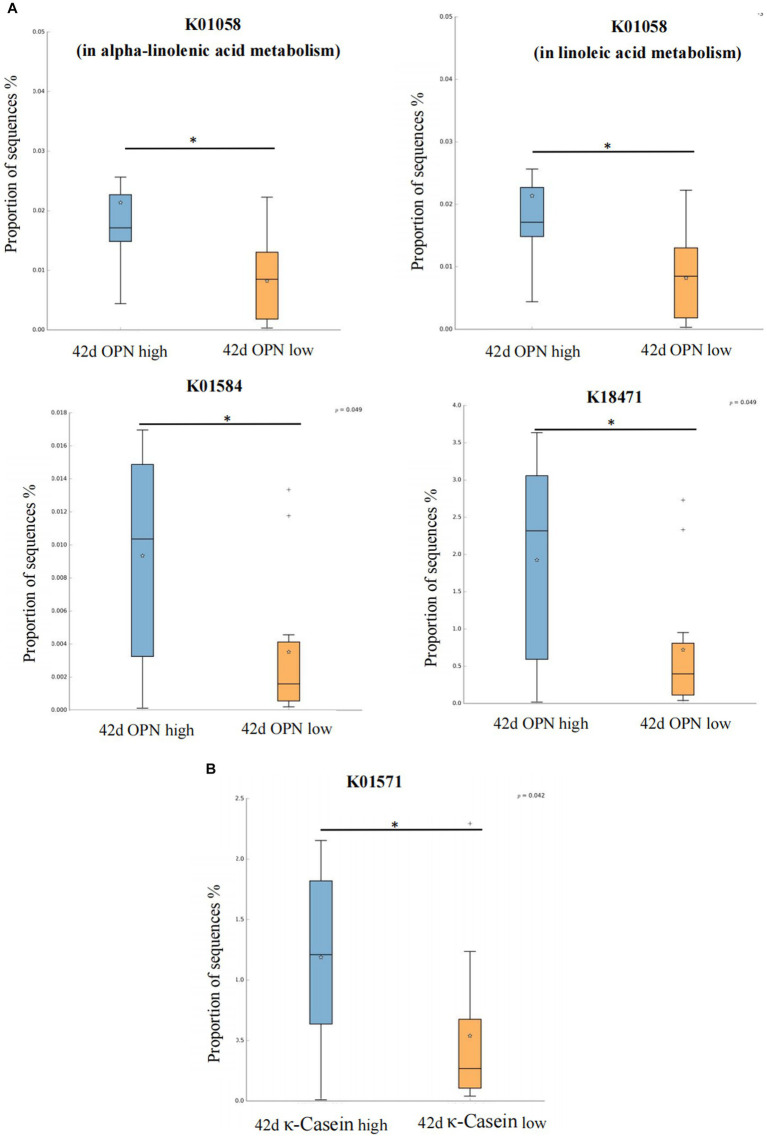
Kyoto Encyclopedia of Genes and Genomes Orthology (KO) analysis of significant differences for human milk proteins. The KO analysis of breast milk **(A)** OPN and **(B)** κ-casein at 42 days, of the low and high content groups. Orange and green indicate low and high content, respectively. The horizontal coordinate represents the different protein groups, and the vertical coordinate represents the abundance of the corresponding differential KO, which K01058 corresponds to phospholipase A1/A2 (EC:3.1.1.32 3.1.1.4), K01584 corresponds to arginine decarboxylase (EC:4.1.1.19), K18471 corresponds to methylglyoxal reductase (EC:1.1.1.-), K01571 corresponds to oxaloacetate decarboxylase (Na + extruding) subunit alpha (EC:7.2.4.2). *p < 0.05.

We also analyzed the KO corresponding to the above metabolic pathways. For OPN at 42 days, the most significant corresponding signaling pathways were alpha-linolenic acid, linoleic acid, arginine and proline, and propanoate metabolism, which involved 26, 25, 107, and 96 related KO, respectively. When we compared these KO with the 6,226 KO annotated with the microbiota data from this study, we found three KO under alpha-linolenic acid metabolism, three KO under linoleic acid metabolism, 60 KO under arginine and proline metabolism, and 68 KO under propanoate metabolism. We used the Wilcoxon test to compare the relative KO abundance between the high and low OPN content groups. Only the functional relative abundance of K01058 [in alpha-linolenic acid and linoleic acid metabolism, which corresponds to phospholipase A1/A2 (EC:3.1.1.32 3.1.1.4)] was significantly higher in the high OPN content group; K01584 [which corresponds to arginine decarboxylase (EC:4.1.1.19)] in arginine and proline metabolism was significantly higher in the high OPN content group. Only the functional relative abundance of K18471 [which corresponds to methylglyoxal reductase (EC:1.1.1.-)] in propanoate metabolism was significantly higher in the high OPN group (Figure 4A).

For κ-casein at 42 days, the most significant signaling pathway was pyruvate metabolism, which involved 133 related KO. When we compared these KO with the 6,226 KO annotated with the microbiota data from this study, we found 92 KO under pyruvate metabolism. When comparing the relative KO abundance between the high and low κ-casein groups, only K01571 [which corresponds to oxaloacetate decarboxylase (Na + extruding) subunit alpha (EC:7.2.4.2)] was significantly higher in the high OPN group (Figure 4B).

## Discussion

In this prospective study, we examined nine proteins that show a significant biological function in infants’ early development and further performed correlation analyses to clarify the association of breast milk proteins with the infant gut microbiota at different time points. To the best of our knowledge, we are the first group to report that different levels of certain proteins in human milk are significantly correlated with the specific microbial species of the infant gut microbiota, particularly *P. distasonis* and *C. butyricum*. KEGG pathway analyses revealed that these proteins may contribute to alter -linoleic acid, alpha-linolenic acid, propionic acid, and pyruvate metabolism by mediating the infant gut microbiota.

### The association of breast milk proteins and the infant gut microbiota

We found that the composition of the infant gut microbiota could be significantly correlated with breast milk proteins, especially OPN, a multifunctional protein with biological activity (Si et al., 2020). Previous reports have mainly focused on the immune function of OPN, but the mechanism is not clear. Researchers found that OPN could reduce the colonization of Lactobacillus in the intestine of mice and promote the abundance of *Dorea*, which could further regulate metabolic disorders (Chen et al., 2022; Shi et al., 2022). In our study, at 42 days, the breast milk OPN content was positively correlated with the abundance of *P. distasonis*. This bacterium is one of the core microbiotas of the human body; it is considered a “second-generation probiotic” and plays an important role in maintaining and protecting intestinal health and the gut barrier (Wang et al., 2019). Koh et al. found that *P. distasonis* is associated with increased expression of the tight junction protein occludin, indicating that this species plays a protective role in the maintenance of the intestinal epithelial (Koh et al., 2020). It also should be noticed that the effects of early breast milk OPN may last for several months, as there was still a positive correlation between the breast milk OPN content at 42 days and *P. distasonis* at 3 months; however, there was no such correlation when comparing the breast milk OPN content and the microbe at 3 months. Considering that the breast milk OPN content declined at 3 months, we suggest that early breast milk OPN plays a more important role in regulating the gut microbiota. At 3 months, OPN was positively correlated with *E. limosum*, which can produce butyrate along with acetate, as the main fertilization end-product from methanol. Kanauchi et al. found that *E. limosum* can reduce colitis, and the metabolite butyrate can enhance the integrity of the mucosa and show anti-inflammatory regulation on the intestinal mucosal system through Toll-like receptor 4 (TLR4) (Kanauchi et al., 2006). There was a similar regulatory effect for lactalbumin at 42 days, which was positively correlated with *P. distasonis*. Previous reports on lactalbumin have pointed to improved early immunity and intestinal mucosal barrier. We speculate that lactalbumin and OPN might jointly affect *P. distasonis* early in life to regulate infant immunity.

At 42 days, we also found that milk κ-Casein was positively correlated with *C. butyricum*, which is a typical intestinal symbiotic bacterium. It has a strong butyric acid production capacity (Stoeva et al., 2021). The final metabolite butyricum, a short-chain fatty acid, can affect the permeability of the colon epithelium and reduce the production of inflammatory factors, which are essential for early intestinal immune system development. Previous studies have also shown that κ-casein has a powerful function in immune regulation (Lalor and O'Neill, 2019). The findings of this study provide a theoretical basis for that the κ-casein might exhibit the certain effects on protecting the intestinal barrier in early childhood, which benefit immunity. However, we found no significant κ-casein functional pathways enriched at 3 months, indicating that the early κ-casein breast milk content may be more important for early microbiota colonization.

In addition, the sIgA content in human milk at 42 days was positively correlated with *V. parvula*. Recently, researchers have reported the ability of sIgA to regulate microbial colonization and the infant immune response (Li et al., 2020; Donald et al., 2022; Pabst and Izcue, 2022). Moreover, *V. parvula* plays a protective and helpful role in the early development of a child’s immune system (Matera et al., 2009). Epidemiological studies on infants have shown that the presence of *V. parvula* was negatively correlated with asthma and bronchiolitis (Romero-Espinoza et al., 2018). Our results suggest that one of the potential mechanisms by which sIgA in breast milk modulates immune function is via its regulation of *V. parvula* during early life.

### Functional analysis based on KEGG

Considering the potential function of the above-mentioned human milk proteins on the gut microbiota of infants, we further explored the metabolic pathways of the above-mentioned different human milk proteomes through KEGG pathway analysis. At 42 days, a higher OPN breast milk content was associated with high expression of K01584 [arginine decarboxylase (EC: 4.1.1.19)] in arginine and proline metabolism; K18471 [methylglyoxal reductase (EC: 1.1.1. -)] in propanoate metabolism; and K01058 [phospholipase A1/A2 (EC: 3.1.1.32 3.1.1.4)] in alpha-linolenic acid and linoleic acid metabolism. Arginine decarboxylase can decompose arginine into spermine and participate in arginine metabolism (Maruri-López and Jiménez-Bremont, 2017). As a conditionally essential amino acid, arginine plays an important role in regulating the physiological activities of intestinal epithelial cells. Ge et al. found that arginine can protect cells from apoptosis caused by oxidative damage induced by lipopolysaccharide (LPS), increase the vitality of intestinal epithelial cells, promote cell division, facilitate intestinal barrier and immune maturation, and reduce the expression of inflammatory factors (Ge et al., 2022). At present, key proteins such as OPN found in this study have been added to infant formula as additive milk powder raw materials. Some intervention studies have shown that adding OPN to infant formula has many beneficial effects, such as promoting the proliferation of infant immune cells, and reducing inflammation (West et al., 2017). Combined with the results of this study, we speculate that its mechanism may be related to the effect of OPN on the gut microbiota and the regulation of the arginine metabolism pathway. At present, there is no report on the relationship between OPN and arginine metabolism. Although our study has revealed for the first time that the OPN content in human milk may be correlated with arginine metabolism in infants, this finding should be verified in future studies. For those children who cannot be breastfed, further understanding of the function of breast milk components will be helpful in designing infant formula that could stimulate breast milk functions. Methylglyoxal reductase is involved in the reduction of propanoate, but its biological function is still unclear. We also found that a certain amount of OPN in human milk may have a potential role in regulating lipid metabolism. For example, phospholipase A1/A2 can catalyze the synthesis of phospholipids rich in linoleic acid and alpha-linolenic acid, which then participate in lipid-related pathways. The results suggest that OPN contributes to biological functions besides the immune system and the intestinal barrier. This eventuality requires further exploration.

At 42 days, κ-casein was related to K01571 in pyruvate metabolism, which corresponds to oxaloacetate decarboxylase (Na + extruding) subunit alpha (EC: 7.2.4.2). Oxaloacetate decarboxylase catalyzes the decarboxylation of oxaloacetic acid to form pyruvic acid, which generates acetyl CoA under the action of pyruvate dehydrogenase, and then butyric acid is synthesized in two steps (Vital et al., 2014). The first step is the conversion of acetyl CoA to butyryl CoA under the catalysis of a series of enzymes. The second step is that butyryl CoA generates butyric acid under the action of the butyric acid kinase (BK), which is the traditional pathway of butyric acid production. In recent years, researchers have found that butyric acid–producing bacteria isolated from gastrointestinal tracts do not have BK producing activity, but could use acetic acid as a carbon source to produce butyric acid under the action of butyryl CoA-acetic acid CoA transferase (Duncan et al., 2002). *Clostridium butyricum* mainly produces butyric kinase through pyruvate, and butyric acid, the main final product of fermentation, affects immune homeostasis and the intestinal barrier in various ways (Stoeva et al., 2021). Our findings suggest that the immune-boosting function of κ-casein may be achieved by regulating the gut microbiota and further influencing the pyruvate metabolism pathway.

### Strengths and limitations

Our study has several strengths. We examined mother–infant pairs to explore the role of breast milk proteins on infant intestinal health. Using a prospective study design, we comprehensively considered the dynamic influence of nine different functional proteins on the gut microbiota, and explored possible functional pathways in combination with KEGG analysis to determine the mechanisms with which breast milk proteins are involved.

There are four main limitations to our study. First, as a pilot study, the sample size was limited. Future studies with a larger sample size are required to confirm the results in current study. Second, although we conducted partial correlation analysis to adjust certain confounding factors, there might be other unknown remaining confounders that could influence the results, such as raising pets in house and using sanitizer. Third, due to a limited budget, nine proteins were analyzed in this study. These studied proteins were chosen because they were recently discovered as functional proteins and because of their reported role in early development, and there were limited literature on their effects on gut microbiota. However, breast milk contains many other proteins that might also influence health by regulating the microbiota, and a joint effect may exist for these proteins. Fourth, there is a lack of analysis of breast milk microbiota, which limited the interpretation of the results.

## Conclusion

Based on 16S rRNA sequencing analysis, we have shown that the contents of specific proteins in human breast milk, such as OPN and κ-casein, are closely related to the abundance of specific intestinal microorganisms in infants, such as *P. distasonis* and *C. butyricum*. Proteins in early breast milk had a more significant effect on the gut microbiota. We also identified the potential pathways of some types of proteins associated with the expression level of certain enzymes, which corresponds to the potential regulation of amino acid, pyruvate, and lipid metabolism. These findings may explain the function of proteins in early immune development, indicate the potential extended function of breast milk protein and support the application of functional proteins used in infant formula. Additional prospective and mother–infant matching studies are needed to clarify the pathways related to human milk proteins, the gut microbiota of infants, and infant health, and to explore their mechanisms.

## Data availability statement

The datasets presented in this study can be found in online repositories. The names of the repository/repositories and accession number(s) can be found in the article/Supplementary material.

## Ethics statement

All participants gave written informed consent. The protocol was approved by the Ethics Committee of Tsinghua University (project no. 20210126). The studies were conducted in accordance with the local legislation and institutional requirements. Written informed consent for participation in this study was provided by the participants’ legal guardians/next of kin.

## Author contributions

MX: conceptualization, data curation, formal analysis, visualization, and writing-original draft. MX, DL, YaY, SD, HL, HY, XS, XN, YuY, CY, IS, and AZ: investigation. MX and AZ: methodology. AZ: project administration and supervision. IS and AZ: writing-review and editing. All authors contributed to the article and approved the submitted version.

## Funding

This work was supported by National Center of Technology Innovation for Dairy and Inner Mongolia Dairy Technology Research Institute (Huhhot Science & Technology Plan, no. 2020-Ke Ji Xing Meng-National Innovation Center-3 project), National Natural Science Foundation of China (grant no. 82273619) and China Medical Board (grant# 21–417).

## Conflict of interest

YaY, SD, and IS were employed by Inner Mongolia Yili Industrial Group, Co. Ltd.

YaY, SD, HL, and IS were employed by Inner Mongolia Dairy Technology Research Institute Co. Ltd.

The remaining authors declare that the research was conducted in the absence of any commercial or financial relationships that could be construed as a potential conflict of interest.

## Publisher’s note

All claims expressed in this article are solely those of the authors and do not necessarily represent those of their affiliated organizations, or those of the publisher, the editors and the reviewers. Any product that may be evaluated in this article, or claim that may be made by its manufacturer, is not guaranteed or endorsed by the publisher.
