# Supplementary material for: Functional proteins in breast milk and their correlation with the development of the infant gut microbiota: a study of mother-infant pairs
Source: Front Microbiol. 2023 Sep 13;14:1239501. doi: 10.3389/fmicb.2023.1239501 (PMC10524269; doi:10.3389/fmicb.2023.1239501)
Supplement: Supplementary file 1 [file Data_Sheet_1.PDF]

SUPPLEMENTARY MATERIAL

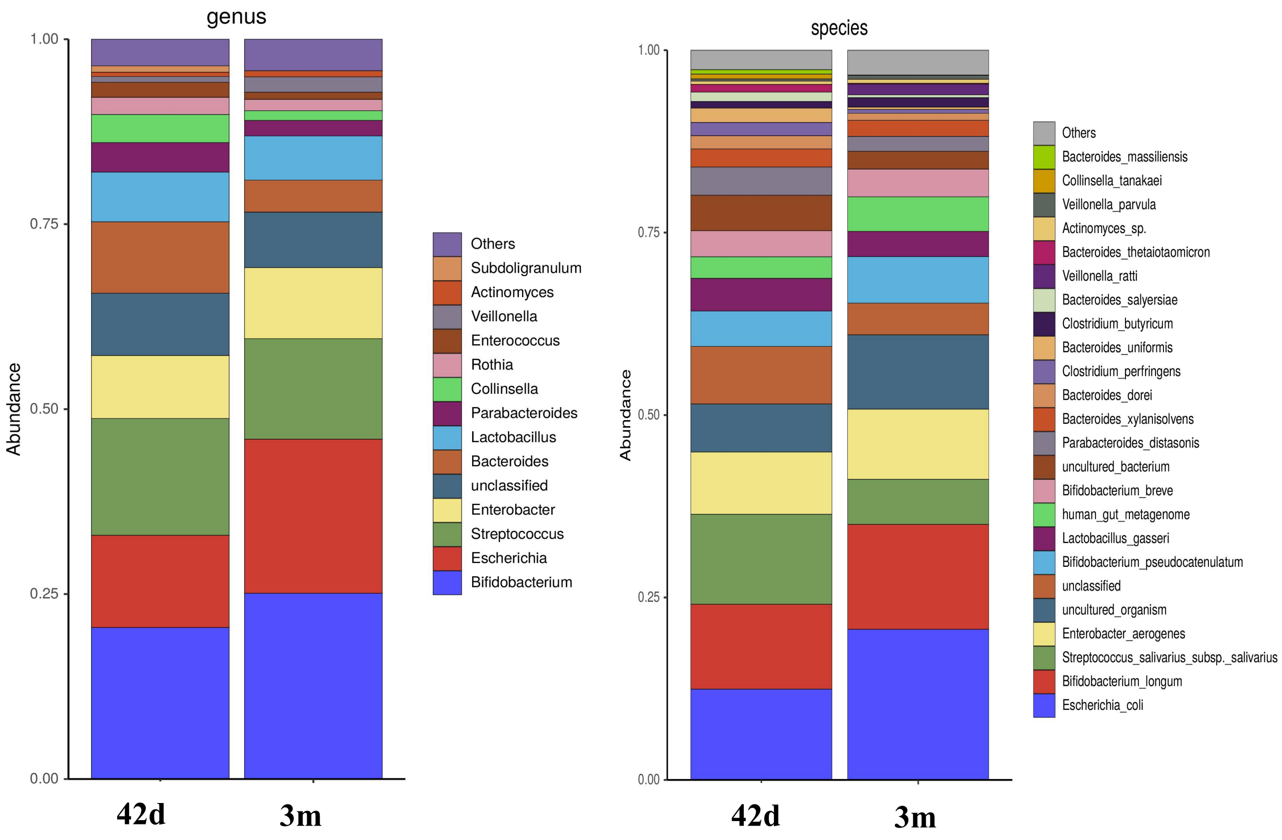

Figure S1 Histogram distribution of intestinal microflora at genus and species levels in infants on the 42 day and 3 months

42d breast milk and 42d infant gut flora

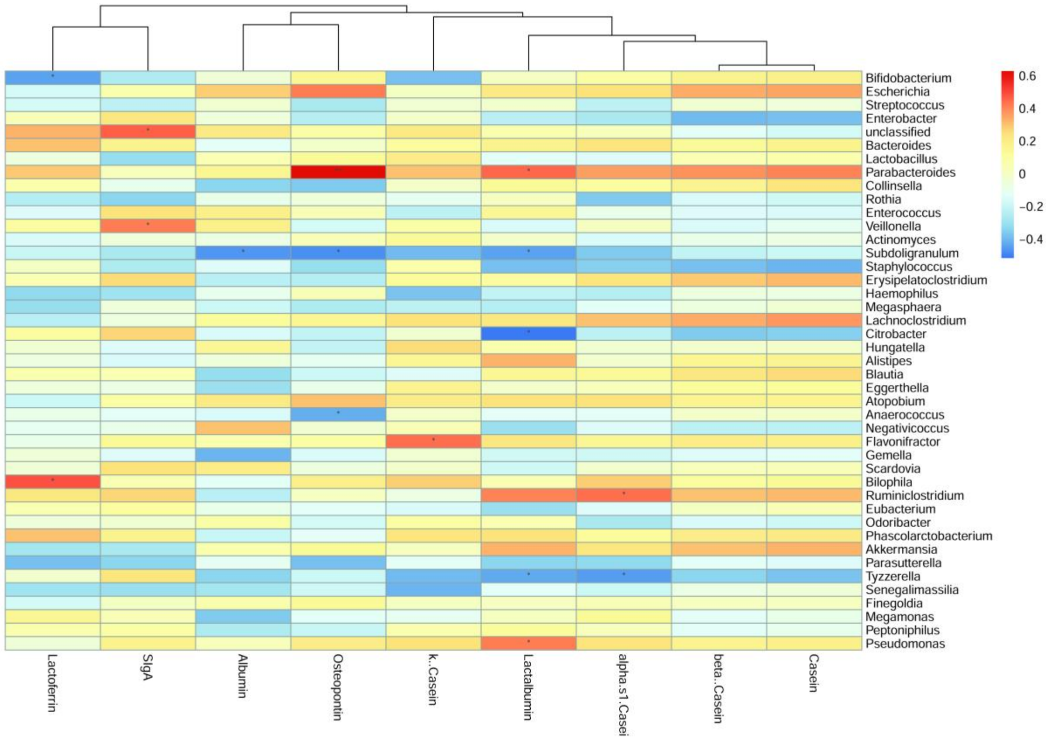

42d breast milk and 3m infant gut flora

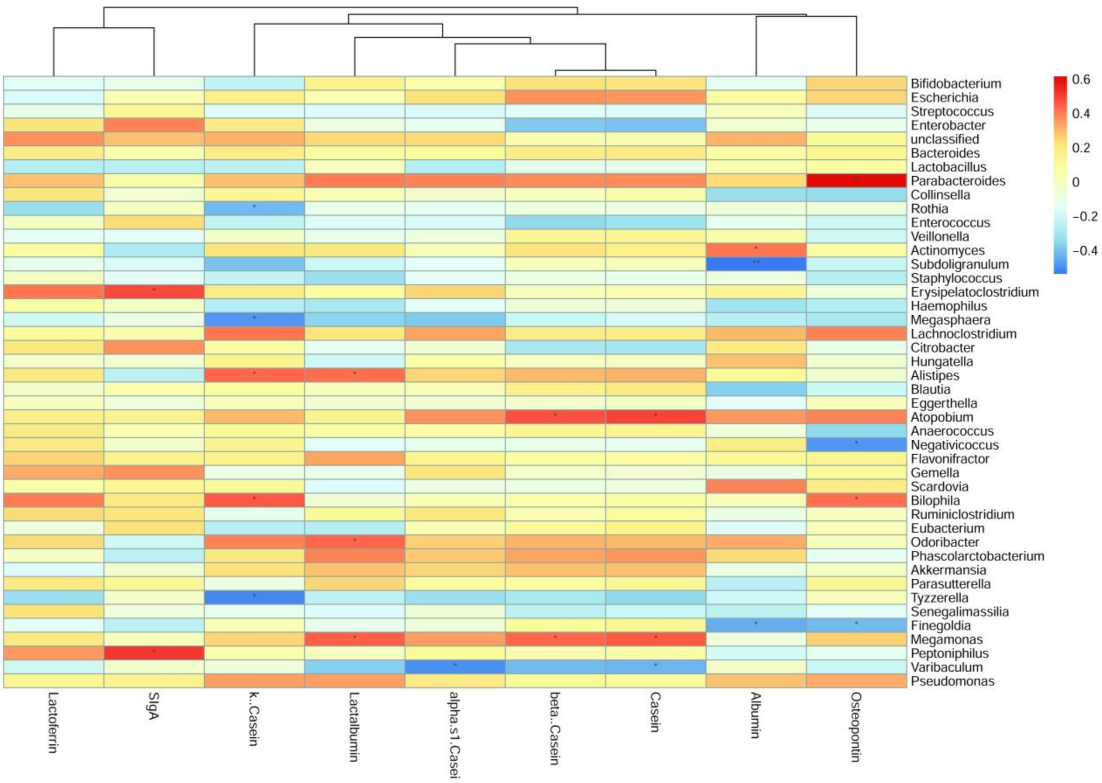

3m breast milk and 3m infant gut flora

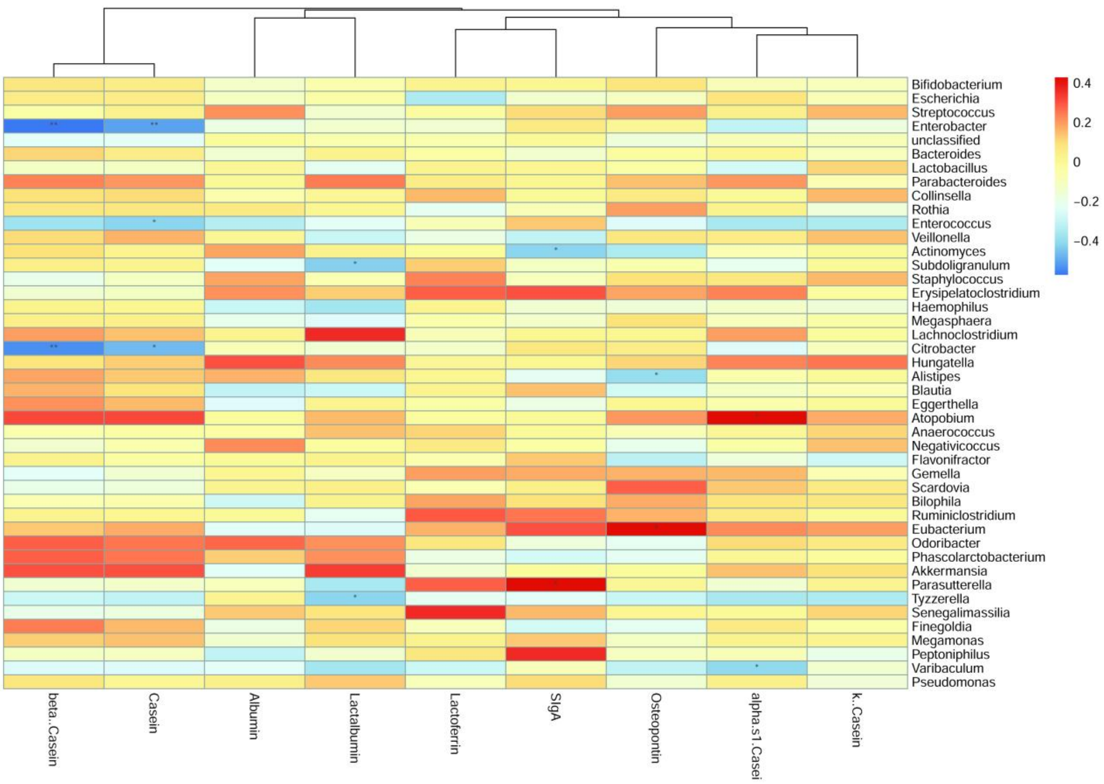

Figure S2 **Correlation heatmap of breast milk proteins with the top 40 most-abundant infant gut microbiota at the genus level based on Spearman analysis.**

42d breast milk and 42d infant gut flora

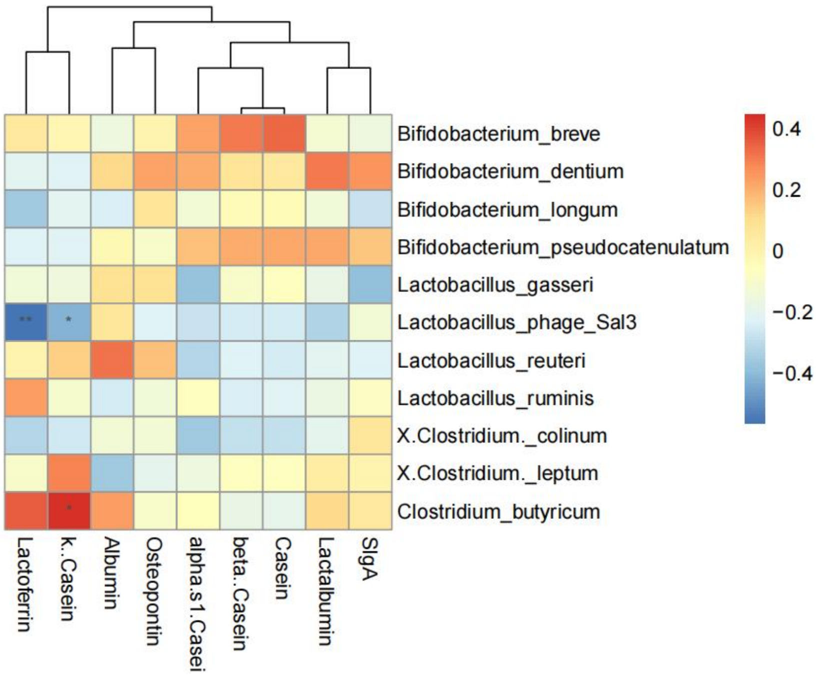

42d breast milk and 3m infant gut flora

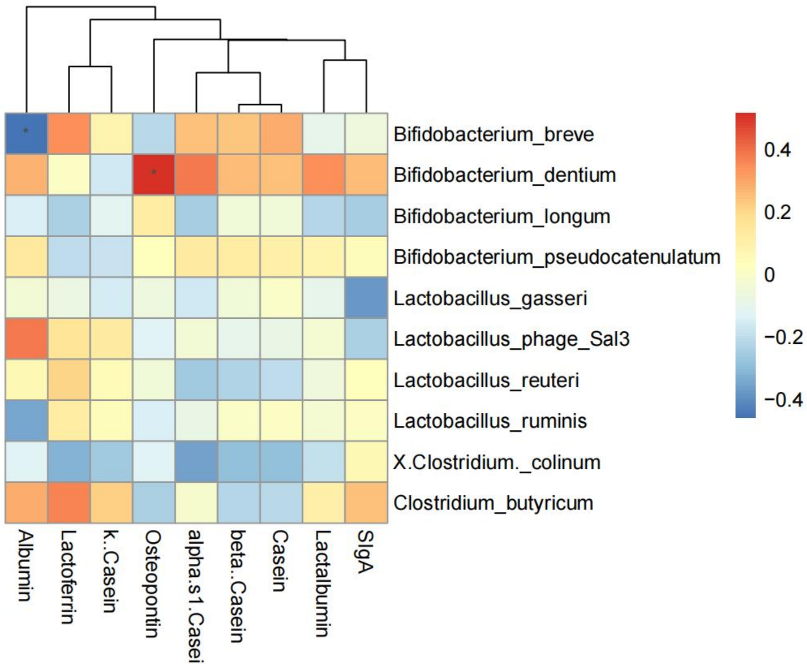

3m breast milk and 3m infant gut flora

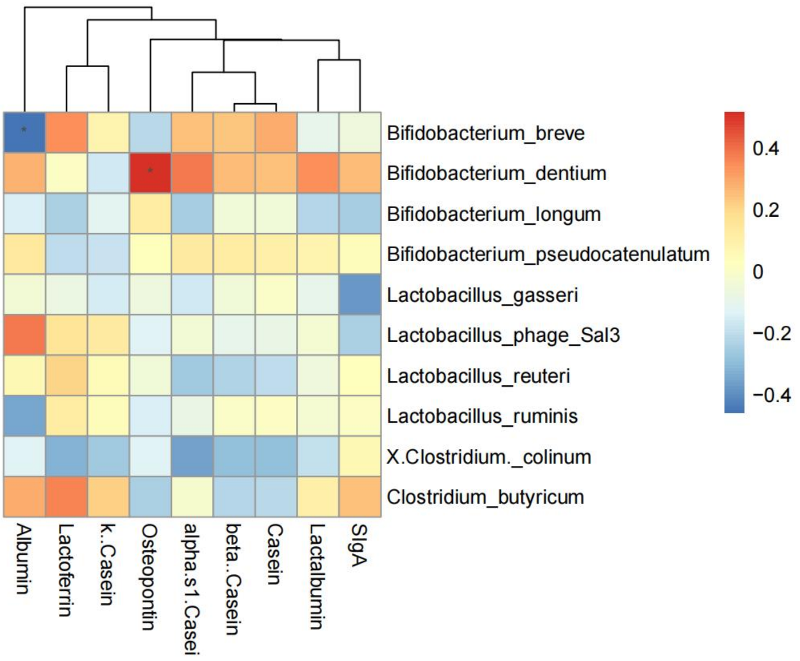

Figure S3 Correlation heatmap of breast milk proteins with the *Bifidobacterium* and *Lactobacillus* of infant intestinal flora based on Spearman analysis.

42d breast milk and 42d infant gut flora

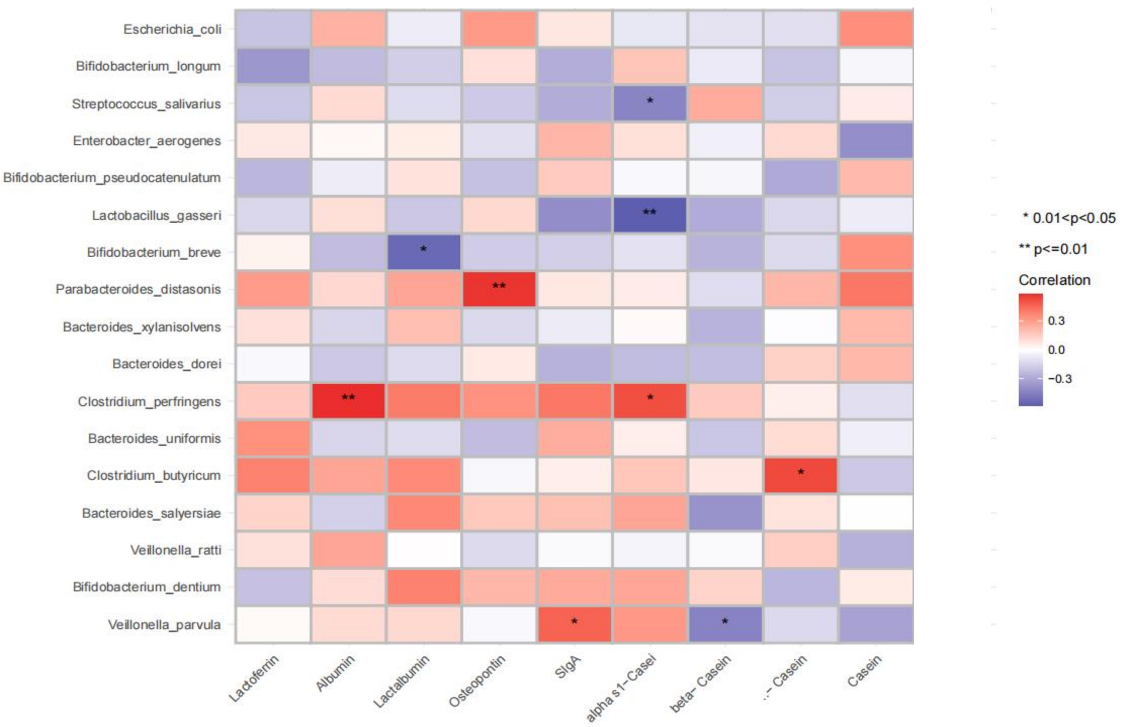

42d breast milk and 3m infant gut flora

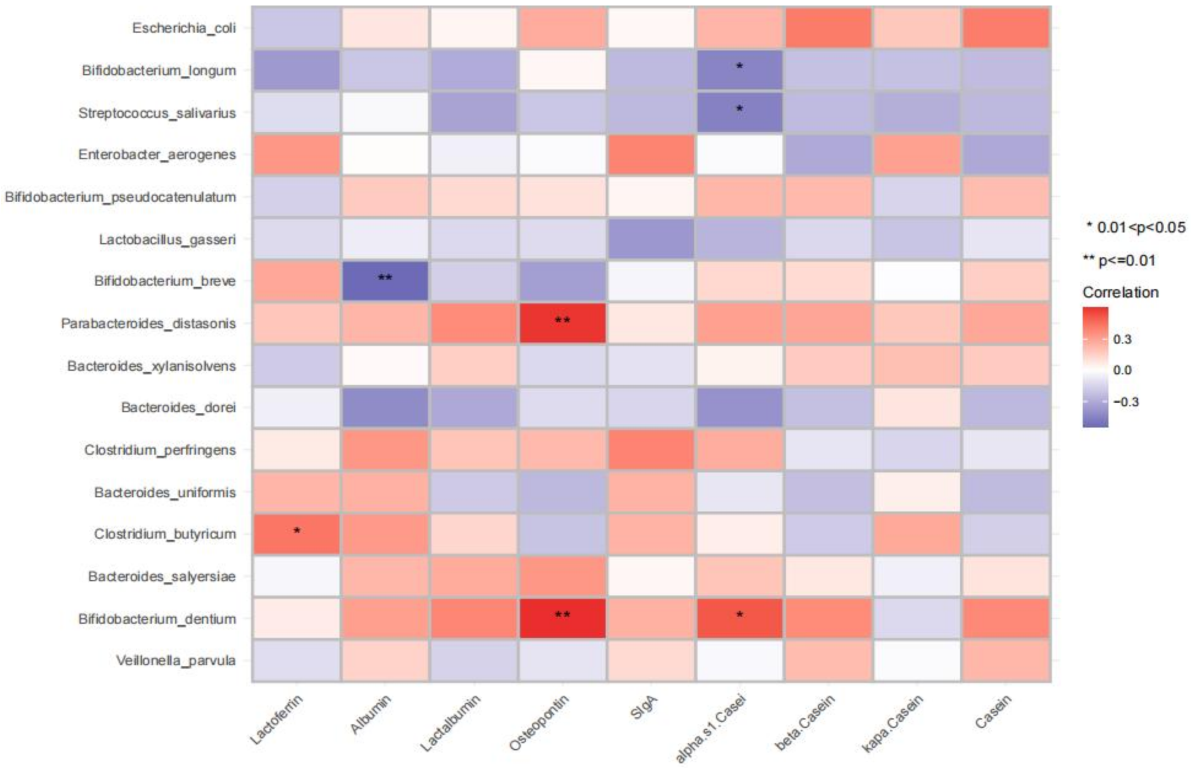

3m breast milk and 3m infant gut flora

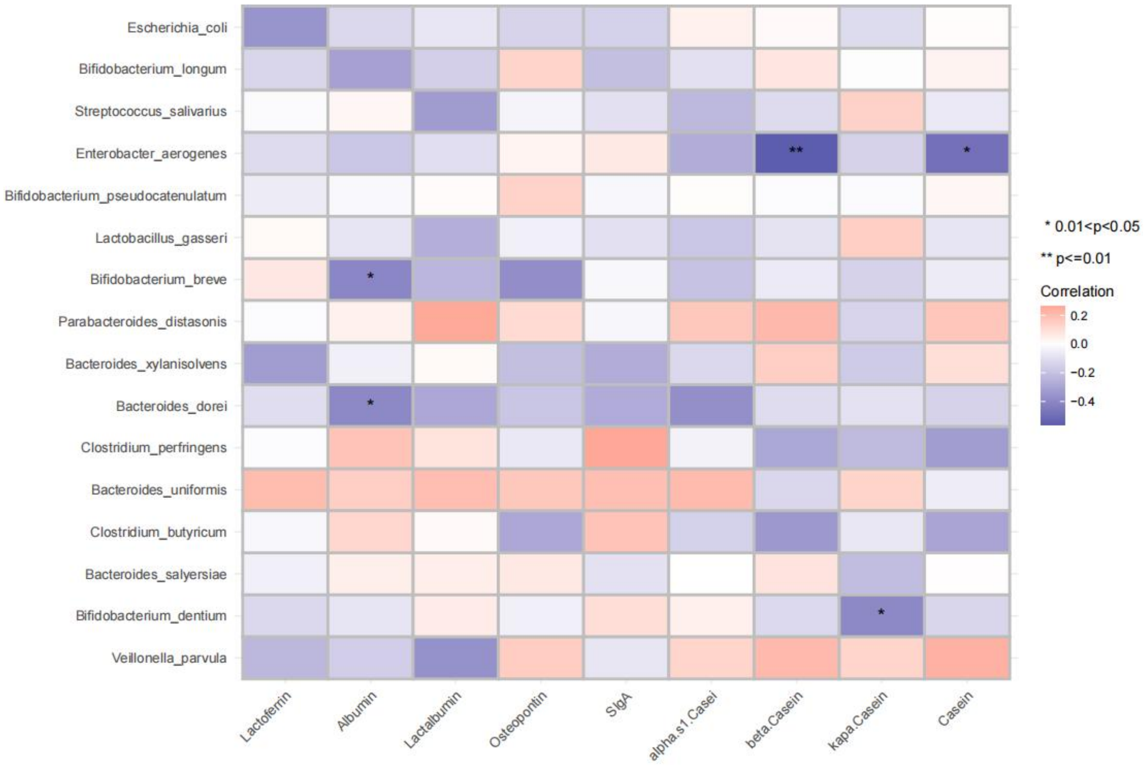

Figure S4 Correlation heatmap of breast milk proteins with the top 40 most-abundant infant gut microbiota at the species level based on partial correlation analysis after controlling delivery mode

Table S1A. Proteins content in breast milk with different delivery mode.

Protein (mg/100g)	42d		P-value*	3m		P-value*
	Vaginal delivery	Cesarean section		Vaginal delivery	Cesarean section	
Lactoferrin	105.5±33.7	88.0±22.8	0.16	82.3±30.0	64.8±12.0	0.24
Albumin	29.1±6.1	26.8±5.8	0.31	25.2±6.1	22.8±5.35	0.68
α- Lactalbumin	386.6±47.3	368.3±46.3	0.44	298.3±35.0	264.6±42.8	0.54
Osteopontin	25.4±4.2	22.1±5.9	0.18	19.5±3.3	16.9±3.4	0.44
SIgA	37.9±14.3	40.0±12.1	0.90	34.8±14.5	31.8±10.3	0.76
αs1-Casei	85.1±12.5	69.9±12.8	0.06	64.9±12.8	53.9±4.5	0.25
β- Casein	411.7±88.6	351.8±52.2	0.12	352.1±87.8	296.1±36.3	0.33
κ- Casein	32.3±6.3	29.5±5.3	0.30	25.1±4.5	21.2±4.8	0.35
Casein	529.2±97.0	448.5±57.1	0.09	439.8±97.2	371.3±42.6	0.33

*Continuous variables were presented as the Mean ± SD. Significant differences between groups were analyzed by Wilcox test.

Table S1B. Proteins content in breast milk with different postpartum weight retention.

Protein (mg/100g)	3m			P-value*
	<3	3-5	>5	
Lactoferrin	81.8±33.0	67.5±32.5	77.9±19.7	0.52
Albumin	27.3±8.7	21.8±4.7	23.9±3.1	0.31
α- Lactalbumin	326.3±28.1	270.1±37.2	269.4±29.8	0.83
Osteopontin	19.2±2.8	17.9±4.9	18.7±3.4	0.63
SIgA	30.4±9.5	35.4±19.8	35.5±12.5	0.47
αs1-Casei	66.5±6.8	61.7±13.4	57.8±13.6	0.38
β- Casein	392.3±34.4	289.9±86.9	316.0±79.5	0.47
κ- Casein	26.4±3.9	25.6±4.4	21.3±4.7	0.65
Casein	479.0±45.1	377.3±89.7	395.1±95.2	0.43

*Continuous variables were presented as the Mean ± SD. Significant differences between groups were analyzed by Wilcox test.
